# Quantitative mass spectrometry imaging of drugs and metabolites: a multiplatform comparison

**DOI:** 10.1007/s00216-021-03210-0

**Published:** 2021-03-26

**Authors:** Lieke Lamont, Darya Hadavi, Brent Viehmann, Bryn Flinders, Ron M. A. Heeren, Rob J. Vreeken, Tiffany Porta Siegel

**Affiliations:** 1 Maastricht MultiModal Molecular Imaging (M4i) Institute, Division of Imaging Mass Spectrometry, Maastricht University, 6229 ER Maastricht, The Netherlands; 2 Janssen Research & Development, 2340 Beerse, Belgium

**Keywords:** Mimetic tissue model, Desorption electrospray ionization, MSI comparison, MRM based drug imaging, Absolute quantification

## Abstract

Mass spectrometry imaging (MSI) provides insight into the molecular distribution of a broad range of compounds and, therefore, is frequently applied in the pharmaceutical industry. Pharmacokinetic and toxicological studies deploy MSI to localize potential drugs and their metabolites in biological tissues but currently require other analytical tools to quantify these pharmaceutical compounds in the same tissues. Quantitative mass spectrometry imaging (Q-MSI) is a field with challenges due to the high biological variability in samples combined with the limited sample cleanup and separation strategies available prior to MSI. In consequence, more selectivity in MSI instruments is required. This can be provided by multiple reaction monitoring (MRM) which uses specific precursor ion-product ion transitions. This targeted approach is in particular suitable for pharmaceutical compounds because their molecular identity is known prior to analysis. In this work, we compared different analytical platforms to assess the performance of MRM detection compared to other MS instruments/MS modes used in a Q-MSI workflow for two drug candidates (A and B). Limit of detection (LOD), linearity, and precision and accuracy of high and low quality control (QC) samples were compared between MS instruments/modes. MRM mode on a triple quadrupole mass spectrometer (QqQ) provided the best overall performance with the following results for compounds A and B: LOD 35.5 and 2.5 μg/g tissue, *R*^2^ 0.97 and 0.98 linearity, relative standard deviation QC <13.6%, and 97–112% accuracy. Other MS modes resulted in LOD 6.7–569.4 and 2.6–119.1 μg/g tissue, *R*^2^ 0.86–0.98 and 0.86–0.98 linearity, relative standard deviation QC < 19.4 and < 37.5%, and 70–356% and 64–398% accuracy for drug candidates A and B, respectively. In addition, we propose an optimized 3D printed mimetic tissue model to increase the overall analytical throughput of our approach for large animal studies. The MRM imaging platform was applied as proof-of-principle for quantitative detection of drug candidates A and B in four dog livers and compared to LC-MS. The Q-MSI concentrations differed <3.5 times with the concentrations observed by LC-MS. Our presented MRM-based Q-MSI approach provides a more selective and high-throughput analytical platform due to MRM specificity combined with an optimized 3D printed mimetic tissue model.

**Supplementary Information:**

The online version contains supplementary material available at 10.1007/s00216-021-03210-0.

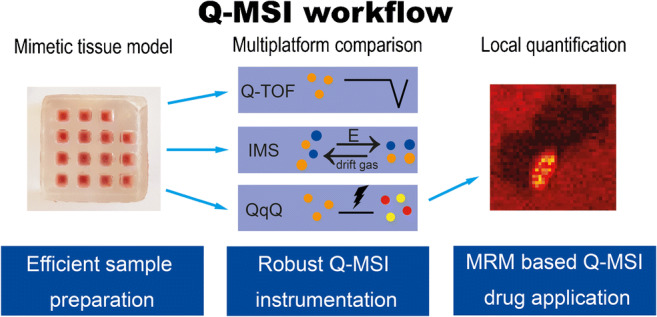

## Introduction

Mass spectrometry imaging (MSI) has proven to be an invaluable tool for the pharmaceutical industry to directly localize drugs and related metabolites from tissue specimens [1, 2]. In the field of drug discovery and development, MSI has been applied in pharmacokinetic and toxicological studies to (amongst others) investigate whether a drug and/or metabolite accumulate in tissue. This (potentially toxicological) accumulation could lead to exclusion of a compound from the drug development pipeline [3]. In addition, MSI plays a key role in pharmacokinetic studies, which are executed to investigate how the drug metabolizes, distributes to the target site, binds to the biological and functional receptor(s) for a certain amount of time at a high enough concentration, and finally releases and excretes within the desired timeframe, all without initiating a toxic effect [4]. Therefore, it is of utmost importance to spatially quantify a possible drug candidate and its metabolites. The ability to provide reliable absolute quantitative information is challenged by numerous factors. These include the lack of sample cleanup or chromatography and, as a result, occurrence of tissue ion suppression and interference of isobaric species in complex biological samples [5, 6].

Quantitative MSI (Q-MSI) has been a field of discussion in the past decade [2, 7, 8]. Researchers have investigated several approaches [9–11] to improve Q-MSI performance [8, 12, 13]. Three common strategies have been applied for the addition of the calibration standards: (i) the tissue extinction coefficient (TEC) model [14], (ii) the dilution series model [15], and (iii) the mimetic tissue model [16]. The TEC approach uses the drug standard (“pseudo” internal standard) sprayed onto a control tissue section. The TEC is a regional correction factor calculated by the intensity of the standard on tissue divided by the intensity of the standard on the glass slide. The TEC can be calculated for different regions within the tissue sample. The sample preparation is quick but the approach lacks the ability to correct for tissue ion suppression and extraction efficiency. The dilution series strategy uses calibration standards spotted onto or below a control tissue section. This way the ion suppression effects are better mimicked but the correction for extraction efficiency is still limited. Recently, the mimetic tissue model was introduced by Groseclose and coworkers [16] and uses tissue homogenates spiked with calibration standards to quantify targeted compounds in the tissue section. Even though it requires more sample preparation, this strategy closely resembles the drug “in-tissue.” Hansen et al. [17] and Barry et al. [18] have both compared the mimetic tissue model and the dilution series model. Much lower absolute intensities and a lower slope value suggest that the mimetic tissue model experiences more tissue ion suppression. As a result, the mimetic tissue model appropriately corrects tissue-specific ion suppression effects and extraction efficiencies better than the dilution series approach. Barry et al. recently reported a revised mimetic tissue model [19] to overcome the time-consuming sample preparation. A quantitative assessment of its performance demonstrated the benefit of their approach [18, 20]. Majority of reported research uses an isotope-labelled standard for correction as the occurrence of tissue ion suppression is not only tissue/organ-specific but also analyte-specific. However, isotope-labelled analogs are rarely available for drug candidates under development or are extremely expensive to obtain.

Several strategies and instruments are available for MSI to overcome the challenge of ion suppression due to the lack of sample cleanup. Derivatization strategies [21] and washing steps, to remove competing sample matrix molecules [22], can be applied complementary to the use of an internal standard (if available). When limited sample preparation strategies are applicable, isobaric interferences can mask the detection of the analyte. High selectivity can separate these isobaric interferences in complex biological samples. This could be achieved through the use of ion mobility separation [23] or high resolving power mass spectrometry combined with imaging [24]. Targeted MSI has also shown advantages for isobaric separation from biological tissue samples [13, 25]. For example, multiple reaction monitoring (MRM) combined with MSI showed promise in several applications in drug distribution studies [26, 27] and endogenous metabolites [28]. Although the use of MRM is already standard practice in the pharmaceutical industry for decades, this is not the case for pharmaceutical MSI. The main advantages of MRM imaging are the high throughput and specific screening of multiple known analytes and a high dynamic range of the instrument [29]. The improved specificity (i.e., near 100% selectivity for one specific analyte) of MRM imaging decreases the interference of background ions and leads to an improved signal-to-noise. This feature of MRM imaging could lead to enhanced performance of Q-MSI. We recently introduced a targeted imaging approach through a combination of MRM with desorption electrospray ionization (DESI) imaging [30]. Although the Q-MSI discussion mainly focuses on MALDI imaging, DESI has established itself as the main ambient ionization source for MSI [31]. DESI uses an electrospray jet to extract and map molecules directly from tissue sections [32]. Unlike MALDI imaging, DESI does not suffer from the signal variation caused by the application of a MALDI matrix prior to MSI analysis. The pharmaceutical industry showed increased interest in DESI imaging [33] for multiple applications [34–37]. A targeted MRM imaging platform could be of great interest for the pharmaceutical industry since the analyte of interest is known prior to analysis and reduces the analysis costs compared to high mass resolving power MSI. We investigate the potential of MRM imaging for quantitative drug imaging in the context of the pharmaceutical demand for Q-MSI technology.

Here, we evaluate different platforms to improve selectivity and sensitivity of Q-MSI. We established a multiplatform comparison amongst different MS detection modes and instruments, followed by a Q-MSI application of two drug candidates in dog liver. First, a mimetic tissue model was adapted to investigate the sensitivity of different platforms using two drug candidates. These samples were analyzed on three different Waters mass spectrometers compatible with the same DESI source and using the same control software: two quadrupole time-of-flight (Q-ToF) instruments (Synapt G2-Si and Xevo G2-XS) and a tandem quadrupole or triple quadrupole (QqQ) instrument (Xevo TQ-S micro), consisting of two quadrupoles and a nonquadrupolar collision cell [38]. We hypothesize that the QqQ has the best analytical performance in MRM mode due to the additional specificity. Limits of detection (LOD), linearity, accuracy and precision from low and high concentrations of quality control (QC) samples were calculated for two drug candidates, to compare the performances of the different platforms and analysis modes. As a proof-of-principle, the mass spectrometer with the best analytical performance was used to map and quantify both drug candidates in dog liver tissues. Quantitative DESI-MSI results were compared to drug levels measured by LC-MS analysis of the same samples.

## Experimental

### Materials and reagents

ULC/MS-grade water (H_2_O), ULC/MS-grade methanol (MeOH), LC/MS-grade ethanol (EtOH), LC/MS-grade xylene, and 99% formic acid (FA) were obtained from Biosolve (Valkenswaard, NL). Gelatin was purchased from Sigma-Aldrich. Microscope glass slides were obtained from Thermo Scientific (Braunschweig, DE).

### Mimetic tissue model preparation and multiplatform comparison setup

**Fig. 1:**
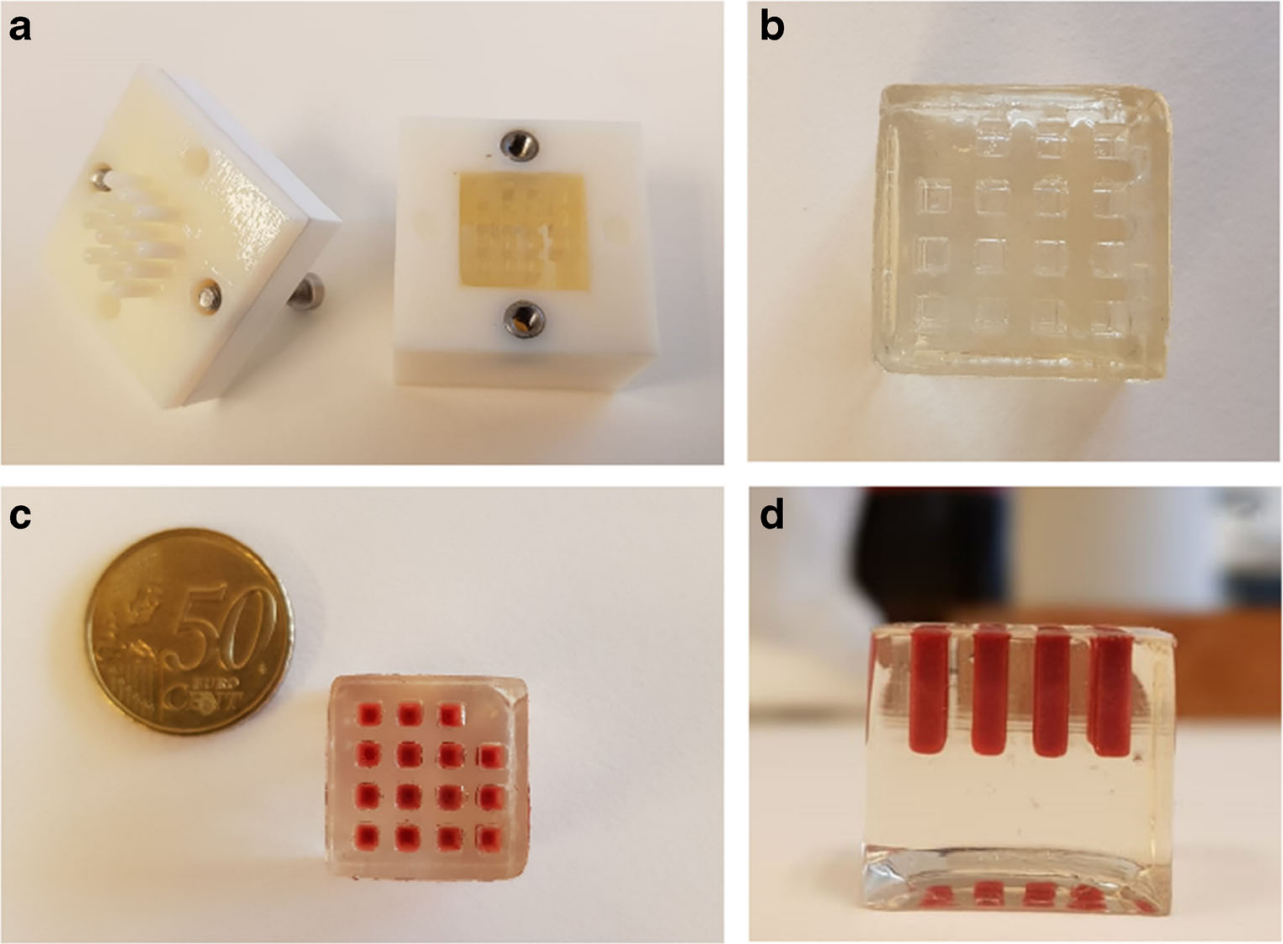
Overview of the mimetic tissue model. Presentation of the 3D printed mold with two sets of screws and 15 pillars in the lid (**a**). Prepared gelatin block after hardening on ice (**b**). The gelatin block filled with spiked tissue homogenate (**c**, **d**) before storage at −80 °C

A mold was printed from VeroWhitePlus RGD835 (Stratasys, DE) using a 3D Objet30 Prime printer (Stratasys, DE). The inside of the mold was covered with a thin layer of clear nail polish (Hema, NL) to prevent adhesion by gelatin. This 3D printed mold (Fig. 1a) was designed with a lid containing 15 squared pillars with 3 × 3 × 8 mm dimensions (*L* × *W* × *H*) screwed onto the outer walls of the mold. A warm 15% gelatin solution was pipetted into the mold and cooled on ice for 30 min to set. After the gelatin was hardened on ice, the two other screws were used to gently remove the lid from the gelatin block. In the multiplatform comparison, chicken liver (Plus supermarket, Maastricht, NL) homogenates were prepared by a mini-bead beater and 1.0 mm glass beads from BioSpec Products (Bartlesville, OK, USA). Chicken liver tissue was used due to the large amount of tissue necessary in the multiplatform comparison. Two drug candidates A and B (Janssen R&D, Beerse, BE) were dissolved in H_2_O and 5 μL was added to 100 mg of the chicken liver prior to homogenization. The volume of the spiked calibration standard was kept 5% (w/w) of the total tissue weight. Final concentrations in the calibration lines were as follows: 12.5, 25, 50, 125, 250, 500, 1250, and 2500 μg/g (C1–C7 calibration levels) tissue. The gelatin block was filled with spiked tissue homogenates in a randomized order to prevent leverage, frozen at −20 °C, and cryosectioned onto glass slides. Glass slides were stored at −80 °C until DESI-MSI analysis. Calibration lines, blank samples (*n* = 3), and quality control (QC) samples at 25 and 1250 μg/g level were prepared in triplicate (*n* = 3 tissue homogenate preparations). The linear dynamic range of the calibration lines covers 3 orders of magnitude for the MS instrumentation/modes comparison. Sensitivity (LOD values), linearity (R^*2*^), QC precision (RSD), and QC accuracy (%) are compared between platforms. Characterization and comparison of the different platform performances are discussed based on the following criteria: (i) QC precision should be ±15% (±20% at lower limit of quantification (LLOQ); defined as SD_blank_ * 5/slope) and (ii) QC accuracy should be −20% to +10%.

### Absolute quantitation in dog liver

Animal studies were conducted in accordance with all institutional and national guidelines for the care and use of laboratory animals. Control and dosed dog liver tissues (beagle) were obtained from Janssen R&D (Beerse, BE). Two drug candidates (A and B) were individually dosed at 65 mg/kg and drug candidate A was also dosed at 15 mg/kg via single oral administration*.* In total, four dogs were included in this proof-of-concept experiment: two animals were dosed 65 mg/kg drug candidate B (dog 1 and 2; *n* = 2), one animal was dosed 65 mg/kg drug candidate A (*n* = 1), and one animal was dosed 15 mg/kg drug candidate A (*n* = 1). All animals were sacrificed 14 days post dose. In vivo, drug B metabolizes into a demethylated metabolite identical to the structure of compound A. Partial molecular structures of both drug candidates as well as their most-likely fragment ion, formed upon collisional activation of the protonated precursor ion, used in MRM are shown in Supplementary Information (ESM) Fig. S1. This research presents an optimized MRM-based Q-MSI approach compared to other MS modes and complemented with an MRM-based proof-of-principle application. Since this research is not a biological investigation of the drug candidates but a technological comparison for Q-MSI, we believe full structural information is not required and, for confidentiality reasons, only the relevant structure is included. A calibration line and quality control (QC) samples (*n* = 3) with drug candidates A and B were prepared in control dog liver using the mimetic tissue model previously described. Fresh-frozen tissues and gelatin block were stored at −80 °C until cryosectioning. A Microtome cryostat (Thermo Scientific, Braunschweig, DE) was used to cryosection liver tissue and the gelatin block into 12-μm-thick slices and subsequently thaw mounted the sections onto microscope glass slides. All glass slides were stored at −80 °C until DESI-MSI analysis.

### Hematoxylin and eosin staining protocol

Tissue slices used for DESI-MSI analysis were stained after completion of the MSI acquisition using a standard hematoxylin and eosin (H&E) staining protocol. Sections were washed in successive EtOH baths (100%, 96%, 96%, 70%, 70%) and deionized H_2_O for 3 min each. Hematoxylin (Merck, Darmstadt, DE) staining was performed for 3 min followed by a gentle 3-min wash with running tap water. Eosin (Avantor Performance Materials B.V., Arnhem, NL) staining was executed for 30 s and washed gently with running tap water for 3 min. The staining was finalized by an EtOH wash for 1 min and a xylene wash for 30 s. The slides were covered by placing coverslips on the stained tissues using Entellan (Merck, Darmstadt, DE). Optical images were acquired using a VENTANA iScan HT scanner (Roche Diagnostics, Indianapolis, IN, USA).

### DESI-MSI instrumentation

DESI-MSI analysis was performed using a DESI source (Waters, Wilmslow, UK) mounted onto three different Waters mass spectrometers: Xevo G2-XS Q-ToF, Synapt G2-Si Q-ToF, and Xevo TQ-S micro QqQ. DESI solvent (MeOH/H_2_O/FA, 98/2/0.1) was supplied to the DESI source by a Waters ACQUITY UPLC M-class binary solvent manager at 2 μL/min. General DESI parameters were as follows: N_2_ nebulizing gas pressure = 4 bar; spray voltage = 3–4 kV; source temperature = 150 °C; sampling cone voltage = 70 V; heated custom-built inlet capillary = 500 °C. All experiments were executed in positive ionization mode. The multiplatform comparison was performed at a pixel size of 100 μm and the quantitative drug application was executed at 50 μm. Different MS modes were used in the sensitivity comparison: MS scan (all mass spectrometers), HDMS and HDMS^E^ (Synapt Q-ToF in ion mobility mode without and with data independent acquisition, respectively), and MRM (Xevo QqQ). All Q-ToF experiments were performed in sensitivity mode. Total scan times were kept constant at 0.986 s/pixel. MRM dwell times were set to 0.247 s/pixel with in total 3 MRM transitions per acquisition: *m/z* 502 → *m/z* 84 (compound A; CE 40 V), *m/z* 516 → *m/z* 98 (compound B; CE 40 V), *m/z* 782 → *m/z* 184 (endogenous lipid; CE 30 V). All MS images were acquired using HDI Imaging (Waters, Milford, MA, USA), MassLynx version 4.1 (Waters, Milford, MA, USA), and Omni Spray 2-D version 2.0.1 (Prosolia, Indianapolis, IN, USA).

### Data processing and visualization

HDI Imaging software (Waters, Wilmslow, UK) was used for visualization of MS images acquired on Xevo Q-ToF and Synapt Q-ToF and select regions of interest (ROIs). We used an in-house written Matlab script (Matlab v. R2015a, MathWorks, Natick, MA, US) for data visualization and ROI selection of the MRM and MS scan data images acquired on the Xevo QqQ. Due to the absence of an isotope-labelled analog of the drug candidates, a homogeneously distributed endogenous lipid (*m/z* 782.6) was used to correct for tissue matrix effects. From each ROI, the extracted MS spectra were summed (100–150 pixels/calibration point) and different peak ratios (analyte-to-lipid) were calculated to build the calibration lines. QuPath software (v0.1.2, The Queen’s University of Belfast, Northern Ireland) was used to select the ROIs from the H&E images and calculate the amount of cells present in the ROI. The H&E images were overlaid with the MSI images by an in-house developed Matlab script and overlapping pixels were extracted. The drug candidate/lipid ratio was calculated after correction for the number of cells detected in the ROI. This workflow can be found in the ESM Fig. S2 [39]. All calibration lines are constructed according to the following steps [40]. A blank sample and 7 calibration points (C1–C7) were measured evenly spaced over 3 orders of magnitude to investigate the linear range of the mass spectrometer. Linear regression analysis is applied and inspected to confirm linearity. Weighted linear regression was evaluated but not beneficial to the linearity. A test sample (dosed dog liver) has been used to confirm that its concentration is within the defined linear range. When a calibration standard concentration was <LOD, this point was excluded from the calibration line.

## Results and discussion

### Optimization of mimetic tissue model

**Fig. 2:**
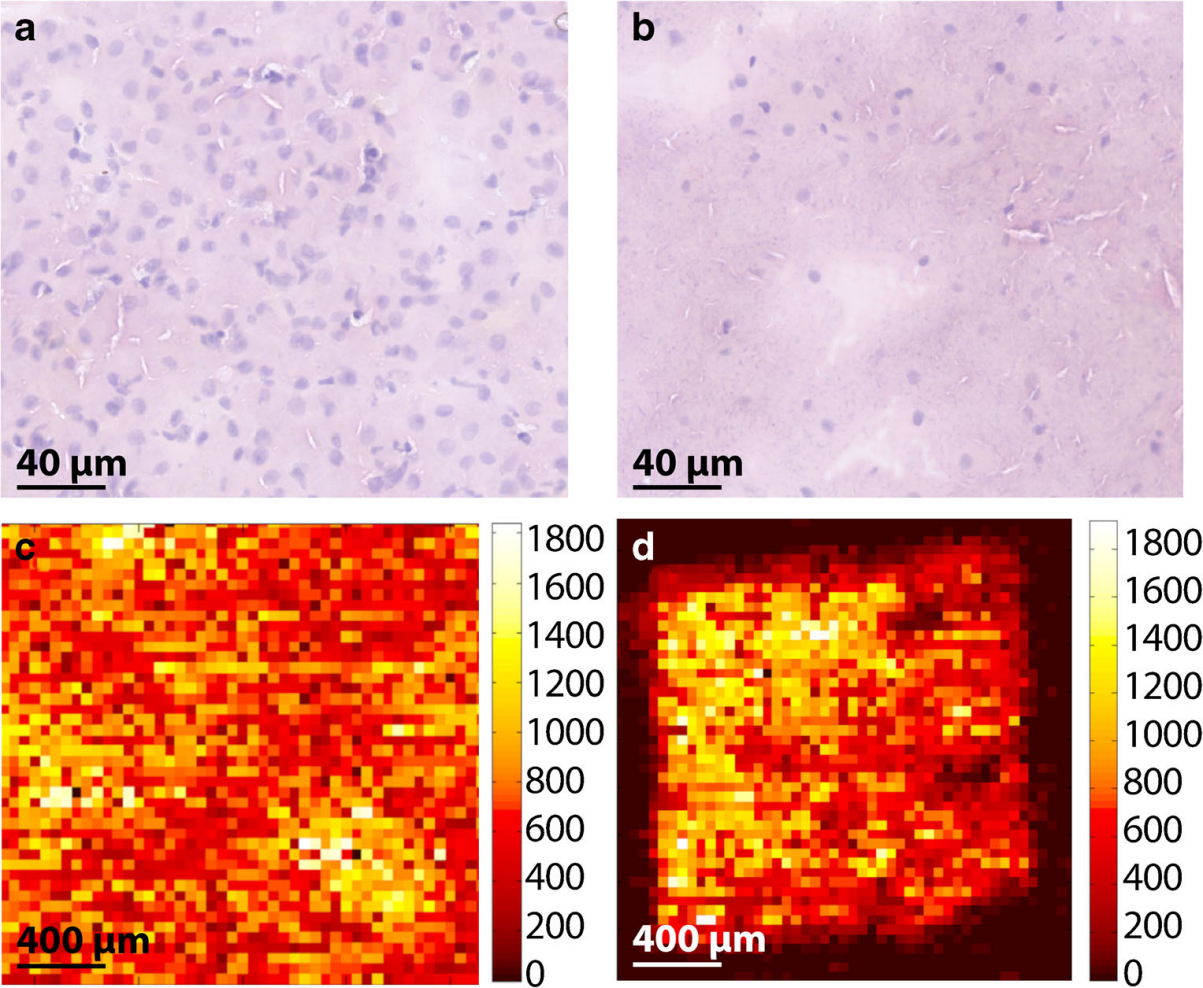
H&E-stained images from liver tissue (**a**) and liver tissue homogenates (**b**). MRM images of *m/z* 782 are shown from liver tissue (**c**) and liver tissue homogenates (**d**). Pixel-to-pixel variation of *m/z* 782 in liver tissue (**c**) and liver tissue homogenates (**d**) is <21% RSD and <24% RSD for *n* = 30 pixels, respectively

The “in-tissue” approach requires a more laborious sample preparation process due to the homogenization step in comparison to the sample preparation of the TEC and the dilution series approaches [41]. In addition, large amount of control tissue is often required to build a mimetic tissue model. Here, we optimized the sample preparation protocol of a mimetic tissue model for high-throughput Q-MSI purposes by designing a 3D printed mold (Fig. 1), used to prepare a gelatin block with a tissue mimetic array. Our approach minimizes the use of control tissue in addition to a high-throughput mimetic tissue model for large-scale drug studies. Gelatin is often used for tissue embedding to assist in cryosectioning fragile or small tissues and is demonstrated to not interfere in the mass spectrum (unlike OCT embedding) [42, 43]. The distance between the pillars of the mold was set at 2 mm to prevent cross-contamination between pillars due to diffusion of molecules from the tissue homogenate into the gelatin. This diffusion was investigated for both drug candidates and the endogenous lipid used for signal normalization (ESM Fig. S3). We noticed minimal diffusion and no cross-contamination of the drug candidates and endogenous lipid. The total dimension of the gelatin block was of 20 × 20 mm^2^ (*L* × *W*; Fig. 1b and c) to fit on a microscope glass slide and to use the additional space for the sample tissue section. The 15 pillars were arranged with one empty corner to keep the orientation clear, which is important after addition of different tissue concentration levels. The volume of the spiked calibration standard was kept 5% (w/w) of the total tissue weight to limit the change in tissue density. Other researchers have added lower percentage of drug standard (<2% by Barry et al. [19]) to the tissue; however, the linear range in the calibration lines and the saturation of the drug candidates required the addition of a higher percentage drug standard. The highly viscous tissue homogenate was aspirated using a disposable syringe to avoid air bubbles in the homogenates and dispensed into the empty pillar using both the syringe and a needle, which resulted in a filled gelatin block (Fig. 1d). The design of our mimetic tissue model allows for high-throughput application due to the large amount of sections that can be obtained from one gelatin block. This is of particular interest for the pharmaceutical industry when performing large-scale drug studies. Figure 2 shows the H&E images of liver tissue before (Fig. 2a) and after (Fig. 2b) homogenization. The morphology of the tissue was lost due to the homogenization step. The addition of 5% of solution containing the spiked standard and the homogenization process itself disrupted the cells, which led to a lower density of intact cells and nuclei. These tissue homogenates were used to mimic the sample tissue and, therefore, simulate an average tissue ion suppression (derived from endogenous cellular compounds) as occurred in actual liver tissue sections [17, 44]. In addition, we compared the distribution of the endogenous lipid (*m/z* 782) before (Fig. 2c) and after (Fig. 2d) homogenization and we confirm that in both cases the lipid intensity and distribution are similar despite cellular disruption caused by the mechanical homogenization process. A mimetic tissue model is challenging to cryosection due to the fragility of frozen tissue homogenate. Inhomogeneity in the cryosectioned calibration line is corrected for by the use of the endogenous lipid unlike other approaches that use a sprayed isotope-labelled standard on top of the mimetic tissue model.

### Multiplatform comparison

The comparison of different mass spectrometers for DESI-MSI of both drug candidates in tissue is shown in Table 1. For each of the MS modes, the limit of detection (LOD), linearity (*R*^2^), QC precision (RSD), and QC accuracy (%) are reported for drug candidates A and B. Table S1 and Fig. S5 (see ESM) complement Table 1 with the intensity ratio and standard deviation of each calibration point used in the multiplatform comparison. Calibration points below the LOD value were excluded from the calibration line. In this paragraph, the results obtained in the multiplatform comparison are compared to the method characterization criteria specified in the “Experimental” section. [45]. While these criteria are inspired by the guidelines set for chromatographic analytical tools applied in toxicological studies, most guidelines are equally applicable to Q-MSI data. The method characterization criteria are used as a point of reference for Q-MSI data.

**Table 1:** Multiplatform comparison of drug candidates A and B

MS instrument	MS mode	Drug candidate A (*n* = 3)				Drug candidate B (*n* = 3)			
		Limit of detection (μg/g)	Coefficient of determination (*R*^2^)	Precision (% RSD at 25*/1250 μg/g)	Accuracy (% at 25*/1250 μg/g)	Limit of detection (μg/g)	Coefficient of determination (*R*^2^)	Precision (% RSD at 25*/1250 μg/g)	Accuracy (% at 25*/1250 μg/g)
Xevo Q-ToF	MS scan	6.7	0.98	3.8/17.8	356/139	2.6	0.98	2.1/18.3	398/100
Synapt Q-ToF	MS scan	36.4	0.92	<LOD/16.5	<LOD/146	4.8	0.90	1.3/16.2	167/115
	HDMS	225.3	0.90	<LOD/17.0	<LOD/103	8.0	0.94	0.1/15.3	621/64
	HDMS^E^	307.3	0.86	<LOD/19.4	<LOD/199	29.7	0.86	<LOD/20.2	<LOD/203
Xevo QqQ	MS scan	569.4	0.93	<LOD/17.7	<LOD/70	119.1	0.95	<LOD/37.5	<LOD/73
	MRM**	*35.5*	*0.97*	*<LOD/2.5*	*<LOD/97*	*2.5*	*0.98*	*8.1/13.6*	*100/112*

*Note that for most of the MS modes, the QC at 25 μg/g level is below LOD

**The best instrument/mode is set in italics

#### Sensitivity

After MSI acquisition of the calibration lines in triplicate, LOD values [46] were calculated by multiplying the standard deviation (SD) of the blank intensity ratios obtained by 3. This intensity ratio was calculated into LOD concentration levels in μg/g tissue by using the slope of the calibration line:

$$ \mathrm{LOD}=\frac{{\mathrm{SD}}_{\mathrm{blank}\left({I}_{\mathrm{drug}\ \mathrm{candidate}}/{I}_{\mathrm{lipid}}\right)}\ast 3}{\mathrm{slope}} $$

The Xevo Q-ToF is a very fast and sensitive instrument and frequently used in combination with DESI in MS scan mode. When comparing the LOD values with those obtained in the three scanning modes on the Synapt Q-ToF, the MS scan on the Xevo Q-ToF is the most sensitive scanning mode for both drug candidates. We investigated all three scanning modes available on the Synapt Q-ToF and all modes showed similar trend for both compounds with MS scan mode being the most sensitive. The application of ion mobility separation is known to improve selectivity due to collisions between the ions and gas molecules and, therefore, eliminates more background ions than analyte ions and enhances *S*/*N* ratios [47]. However, due to these ion-neutral collisions, total ion transmission decreases significantly [48]. For our two drug candidates, we conclude that HDMS^E^ is the least sensitive mode on the Synapt Q-ToF. MS scan mode on the Xevo QqQ gave high LOD value for compounds A and B. The LOD values reported a strong sensitivity gain in MRM mode: 50-fold for compound B and a mere 20-fold for compound A. Compound A was imaged most sensitive in MS scan mode on the Xevo Q-ToF. In summary, distinct specificity enhancement was obtained for compound B in MRM mode versus MS scanning modes. This sensitivity gain by using MRM mode seems to be much larger for compound B than for compound A. The explanation for a poorer sensitivity for compound A in MRM mode versus compound B in MRM mode could be a result of different fragmentation efficiencies. During optimization of the MRM transition, one is always searching for the most abundant fragment ion. However, this is not always a guarantee for the best *S*/*N* ratio for this transition. Therefore, the most abundant fragment ion may not provide the same sensitivity for both compounds. In other words, if the selectivity gain (a higher *S*/*N* ratio due to the observed background) of the MRM transition for compound B (*m/z* 98) is significantly higher than the *S*/*N* of the MRM transition for compound A (*m/z* 84); then, this will result in different detection limits.

#### Linearity

For both compounds A and B, the best linearity was observed on the Xevo Q-ToF in MS scan mode (*R*^2^ > 0.98, Table 1). The significantly poorer correlation was obtained on the Synapt Q-ToF in all modes (*R*^2^ 0.86–0.94). On the Xevo QqQ, the coefficient of determination was poor in MS scan mode (*R*^2^ 0.93) and improved in MRM mode (*R*^2^ 0.97) for compound A. For compound B, linearity values acquired on the Xevo QqQ (*R*^2^ > 0.95) report improvement compared to the Synapt Q-ToF and are comparable to the Xevo Q-ToF. Variability in calibration lines could be limited by including more levels per calibration line, for example, ≥10 calibration levels (instead of ≥7 levels for chromatographic approaches).

#### Precision and accuracy

Spiked QC samples were included in triplicate in the experimental setup to investigate the performance of our Q-MSI workflow. QC samples were prepared at two concentration levels, i.e., 25 and 1250 μg/g tissue. Precision was calculated by relative standard deviations (RSD) based on *n* = 3 replicates. For some MS modes, the QC samples at 25 μg/g were below the observed LOD values and are not reported in Table 1. Precision should be ±15% (±20% below lower limit of quantification (LLOQ); defined as SD_blank_ * 5/slope) [45]. When we compare the precision levels obtained from the different MS modes, the MRM mode is the only MS mode that meets the method characterization criteria for both drug candidates. With regard to accuracy, the criteria is set to −20% to +10% [45]. In our multiplatform comparison, no MS mode meets the method characterization criteria for both drug candidates; however, MRM mode is the closest in analytical performance to the method characterization criteria. The applicability of these criteria to Q-MSI data is debatable due to the higher variability in Q-MSI approaches than in chromatographic strategies, which include the use of internal standards. More QC replicates in the Q-MSI experimental design could be considered to meet the method characterization criteria, for example, *n* ≥ 5, instead of *n* = 3 as used in our experiments.

Overall, one could argue that compound B showed a significantly lower LOD value than compound A in MRM mode. The sensitivity might be better in MS scan mode (Xevo Q-ToF); however, the linearity and the precision and accuracy of high and low QC samples show the best analytical performance in MRM imaging mode compared to all MS scanning modes for both drug candidates. Therefore, we selected the MRM imaging mode for quantitative analysis of the drugs in dog liver samples.

### Absolute quantitation of drug candidate compounds in dog livers

Q-MSI based on our optimized mimetic tissue model allows for high-throughput screening in drug studies that comprise multiple animals at multiple time points. We demonstrate this Q-MSI drug application as a proof-of-concept based on four different dog liver samples. These samples were obtained from a drug development study of two possible drug candidates (compounds A and B) and were analyzed with DESI-MRM. Dogs 1 and 2 are dosed 65 mg/kg B, dog 3 is dosed 65 mg/kg A, and dog 4 is dosed 15 mg/kg A for this experiment.

#### DESI-MSI imaging of dog liver samples

**Fig. 3:**
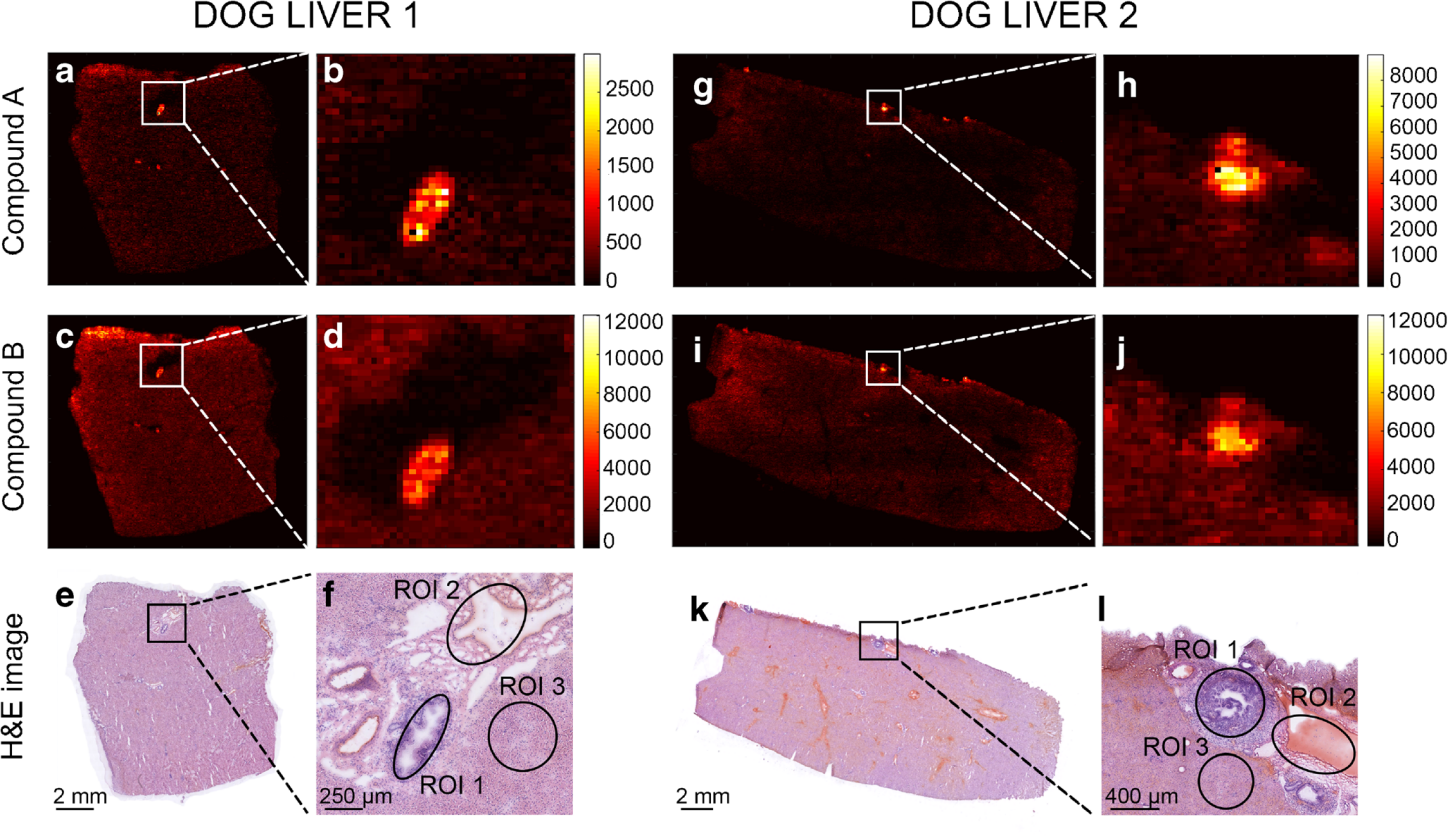
DESI-MRM images obtained from livers of dogs 1 and 2 (dosed with drug candidate B at 65 mg/kg). For dog liver 1, dealkylated metabolite image (**a**, **b**) and drug candidate B (**c**, **d**). Corresponding H&E images (**e**, **f**) show the tissue lesion and define the three selected ROIs. In addition for dog liver 2, dealkylated metabolite image (**g**, **h**) and drug candidate B (**i**, **j**). Corresponding H&E images (**k**, **l**) show the tissue lesion and define the three selected ROIs

**Fig. 4:**
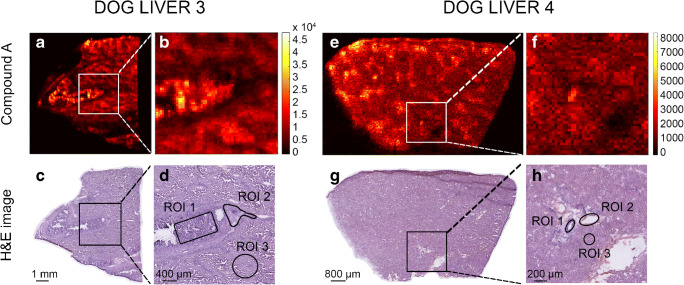
DESI-MRM images obtained from dog livers 3 and 4 of which the animals were dosed drug candidate A at 65 mg/kg and 15 mg/kg, respectively. For dog liver 3, drug candidate images (**a**, **b**) are shown. Corresponding H&E images (**c**, **d**) show the tissue lesion and define the three selected ROIs. For dog liver 4, drug candidate images (**e**, **f**) are shown. Corresponding H&E images (**g**, **h**) show the tissue lesion and define the three selected ROIs

Figure 3 displays the DESI-MRM image data obtained from sections of dog livers 1 and 2 for compound B (Fig. 3c and i). In both dog liver sections, an accumulation of compound B in the bile duct (zoom in Fig. 3d and j) was noticed. The dealkylated metabolite (compound A), also detected in dog livers 1 and 2 (Fig. 3a and g), showed the same accumulation location (zoom in Fig. 3b and h) as drug candidate B. A detailed examination by a trained pathologist of the H&E-stained tissue sections showed that both drug candidates caused histological lesions in the dog livers (Fig. 3e and f for dog liver 1; and Fig. 3k and l for dog liver 2). Figure 4 presents the MRM compound images obtained from dog liver 3 (Fig. 4a and b) and dog liver 4 (Fig. 4e and f) for drug candidate A. The MRM images of the distribution of compound A in dog livers 3 and 4, where the actual compound A is directly dosed to the dogs, did not show the same accumulative pattern in “hot spots” as occurred in dog livers 1 and 2. Q-MSI data is necessary to explain this observational difference between the two sets of dog livers.

#### From pixels to Q-MSI

**Fig. 5:**
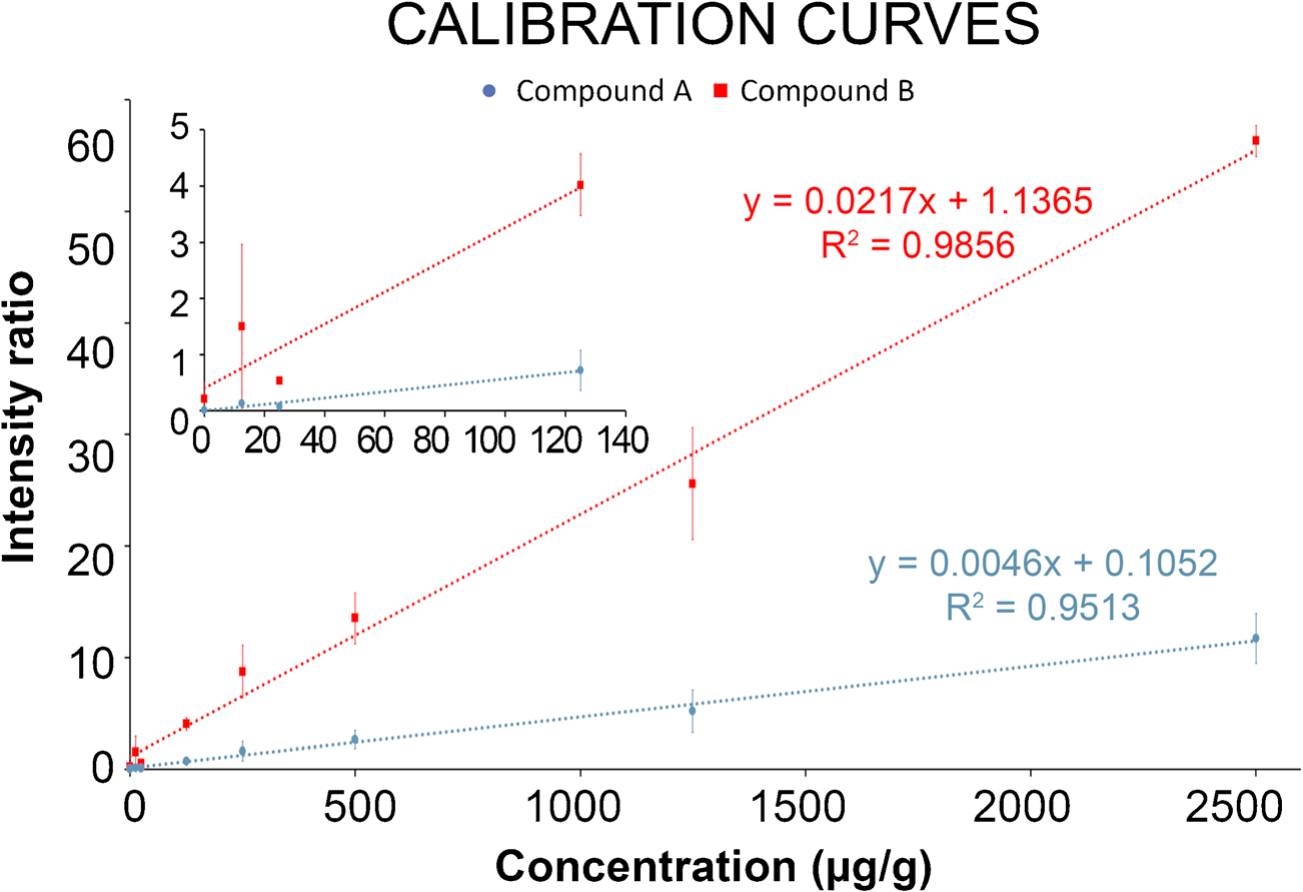
Calibration curves (*n* = 2) of drug candidates A (blue) and B (red) in control dog liver that were used to quantify the three ROIs in the four dog liver tissues. A separate plot depicts the lower range of the calibration curves. Error bars show the standard deviation of the intensity ratios for each calibration level

For the quantitative calculations in the different dog livers, three different regions were highlighted (Figs. 3f, l, 4d and h) and corresponding spectra extracted from the MSI data: (*ROI 1*) tissue lesions in the bile duct and bile duct wall; (*ROI 2*) the connective tissue with part of the blood vessel; (*ROI 3*) the liver parenchyma [49]. In each dog liver tissue, ROI 1, ROI 2, and ROI 3 were extracted in triplicate (*n* = 3). Their H&E-based regions can be found in the ESM (Fig. S4). Some of these extracted ROIs were marked in the H&E images in Figs. 3f, l, 4d, and h for dog livers 1, 2, 3, and 4, respectively. Tissue ion suppression correction is necessary for more accurate quantitative results [50]. Due to the absence of an isotope-labelled analog of the drug candidates, an endogenous lipid (*m/z* 782) was used as correction factor. The endogenous lipid used for correction is homogeneously distributed throughout the tissue homogenates but not throughout the dog liver and, therefore, is not an ideal correction factor. The extraction of pixels that exclusively contain this endogenous lipid was guided by the regional cell count in the dog liver [39]. This workflow is shown in ESM Fig. S2. Figure 5 depicts the calibration line for both drug candidates to obtain the quantitative results in the three ROIs. These calibration lines were constructed in control dog liver with the same linear range as used in the multiplatform comparison (12.5–2500 μg/g) which include the targeted concentration range. With coefficients of correlation *R*^2^ > 0.95, linear calibration lines were constructed and used for quantification of the three ROIs. For drug candidate A, precision levels of QC samples (*n* = 3) were 11% at 25 μg/g and 14% at 1250 μg/g and the accuracy was determined at 91% at 25 μg/g and 94% at 1250 μg/g. For drug candidate B, the calculated precision was 12% at 25 μg/g and 12% at 1250 μg/g. Accuracy was established 94% at 25 μg/g and 93% at 1250 μg/g. The final absolute concentrations quantified in the ROIs are listed in Table 2. In line with the observations from the MRM images, the “hot spot” (e.g., tissue lesion) showed a significantly higher concentration for compound A (metabolite) than the connective tissue/blood vessel (6.6 times higher) and liver parenchyma (2.2 times higher) for dog liver 1. However, this was not the case for dog liver 2 where tissue lesions and liver parenchyma resulted in similar concentrations (lesion 1.02 times higher than parenchyma). Considering that compound A is metabolized from drug candidate B, the concentrations observed in the tissue lesions are lower than those in liver parenchyma for dog liver 1 (1.6 times lower) and dog liver 2 (2.7 times lower). It is clear from the data that for both dog livers 1 and 2, connective tissue/blood vessel resulted in significantly lower concentrations. Considering that dog livers 1 and 2 are biological replicates, a similar trend was observed amongst the three ROIs. A clear difference was observed in the metabolism of the dogs, in which dog 2 metabolized more of the drug candidate (B) into its metabolite (A) than dog 1. The connective tissue/blood vessel and liver parenchyma particularly showed a higher metabolic rate, which confirms the need for multiple animals in large-scale drug studies. The absolute concentration levels of dog livers 3 and 4 showed a factor difference of >5 for tissue lesion (2549 and 465, respectively; factor 5.5) and connective tissue/blood vessel (2302 and 366, respectively; 6.3) which can potentially be explained by the dosage difference between dog livers 3 and 4 (factor 4.3). The observation that dog livers 3 and 4 showed minimal “hot spots” of compound A in the MRM images is confirmed by the quantitative MSI data. All three ROIs yielded similar concentrations. In addition, the differences in absolute concentrations detected in the different tissue regions stress the need for spatial quantitative information that can explain the distribution of drugs and their metabolites in toxicological studies. Regional laser capture microdissection of tissue followed by LC-MS can provide absolute quantification in tissue but is an extremely laborious approach when performed on multiple animals at different time points [51, 52]. Using our mimetic tissue model allows spatial quantitative screening of drugs and metabolites in large-scale toxicology studies without being too time consuming.

**Table 2:** Quantification of drug candidates A and B in three ROIs in four dog liver tissues

		Concentration (μg/g)		
		Tissue lesion	Connective tissue	Liver parenchyma
Dog liver 1 (65 mg/kg dosed B)	A	526 ± 156	79 ± 27	242 ± 59
	B	316 ± 115	172 ± 71	495 ± 78
Dog liver 2 (65 mg/kg dosed B)	A	588 ± 126	136 ± 98	577 ± 227
	B	100 ± 21	58 ± 84	269 ± 103
Dog liver 3 (65 mg/kg dosed A)	A	2549 ± 673	2302 ± 966	>ULOQ
	B	ND	ND	ND
Dog liver 4 (15 mg/kg dosed A)	A	465 (*n* = 1)	366 ± 28 (*n* = 2)	480 ± 50
	B	ND	ND	ND

#### LC-MS comparison

The use of four dog livers from this pharmaceutical study allowed us to compare quantitative DESI-MRM data with earlier obtained LC-MS data. The comparison of Q-MSI data with LC-MS data of the same tissue is challenging because of lacking spatial information from the tissue homogenates analyzed by LC-MS. Barry and coworkers discussed the legitimacy of LC-MS validation of Q-MSI based on three case studies [20]. Only if the MSI image shows a homogenous distribution, LC-MS data is comparable to Q-MSI data. Because our LC-MS data was obtained from assays that homogenize the whole liver tissue, as a consequence, we only compare the quantified MRM data of the liver parenchyma with the LC-MS data since the contribution of the parenchyma to homogenized tissue is by far the largest. Three different regions of interest were extracted from the liver parenchyma (*n* = 3). We only report concentration ranges of the LC-MS data from the dog livers due to confidentiality of the drug candidates. LC-MS concentrations for compounds A and B obtained in dog liver 1 were ~150–220 μg/g and ~120–250 μg/g in dog liver 2. Drug concentrations of compound A found in dog liver 3 were above the upper limit of quantification (<ULOQ) and in dog liver 4 were ~1550–1650 μg/g. A comparison between quantitative LC-MS and quantitative DESI-MRM imaging demonstrated a difference in absolute concentrations of <3.5 times. Tissue heterogeneity, local information which is lost in LC-MS of tissue homogenates, influences the regional signal by the occurrence of tissue ion suppression. The ideal strategy to correct for this tissue ion suppression is the use of an isotope-labelled analog of the drug candidate. In addition, the contribution of the liver parenchyma to a tissue homogenate of the same liver sample is the largest but is not 100%. Therefore, the comparison of LC-MS of homogenates and Q-MSI of liver parenchyma could be inadequate. LC-MS and regional sampling, such as LESA [5] or laser capture microdissection [51], could provide more agreement between Q-MSI and LC-MS as the distribution in tissue is the starting point.

## Conclusions

This work reports an optimized design for mimetic tissue model followed by an analytical assessment of its performance and a proof-of-concept drug application in Q-MSI. Our optimized protocol fits into an “in-tissue” approach and allows Q-MSI analysis of the sample tissue on the same glass slide. The large number of sections that can be obtained from one gelatin block can be used for large-scale drug studies. In addition to this mimetic tissue model, MRM imaging has been investigated in comparison to different MS modes. After evaluation of multiple MS modes, MRM imaging has enhanced the analytical performance of Q-MSI. This is a consequence of the improved specificity obtained from MRM imaging and, therefore, can visualize more targeted analytes without the isobaric interference of background compounds. As a proof-of-concept, Q-MSI drug levels were obtained from four different dog livers and compared with quantitative LC-MS. Although more animals are needed to confirm the obtained concentration levels, significant concentration differences were observed between tissue lesions, connective tissue and blood vessels, and liver parenchyma of the two animals (dogs 1 and 2) that were dosed 65 mg/kg drug candidate B. The two animals (dogs 3 and 4) that were dosed 65 and 15 mg/kg drug candidate A, respectively, did not show this accumulative behavior of the drug candidate in the tissue lesion. The comparison with LC-MS data revealed a concentration difference of <3.5 times between quantitative LC-MS and quantitative MRM imaging for liver parenchyma. The analytical validation of Q-MSI is still challenging and needs to be compared with LC-MS. Our developments contribute to a more selective Q-MSI workflow for drug imaging: an adapted mimetic tissue model for high-throughput toxicological studies and a more sensitive and specific MRM detection to improve biological variability.

## Supplementary information

ESM 1 (PDF 850 kb)
