# Supplementary material for: Quantitative mass spectrometry imaging of drugs and metabolites: a multiplatform comparison
Source: Anal Bioanal Chem. 2021 Mar 26;413(10):2779–91. doi: 10.1007/s00216-021-03210-0 (PMC8007509; doi:10.1007/s00216-021-03210-0)
Supplement: Supplementary file 1 — (PDF 850 kb) [file 216_2021_3210_MOESM1_ESM.pdf]

Supplementary Information

Quantitative Mass Spectrometry Imaging of Drugs and Metabolites: a Multiplatform Comparison

AUTHORS

Lieke Lamont,[a] Darya Hadavi,[a] Brent Viehmann,[a] Bryn Flinders,[a] Ron M. A. Heeren,[a] Rob J. Vreeken,[a,b,*] Tiffany Porta Siegel[a,*]

[a] Maastricht MultiModal Molecular Imaging (M4I) institute, Division of Imaging Mass Spectrometry, Maastricht University, 6229 ER Maastricht, The Netherlands

[b] Janssen Research & Development, B-2340 Beerse, Belgium

* Corresponding authors: Rob Vreeken rvreeken@ITS.JNJ.com ; Tiffany Porta Siegel t.porta@maastrichtuniversity.nl

Table of contents

List of Tables:

Table S1. Intensity ratio and standard deviation of calibration points in multiplatform comparison

List of Figures:

Fig. S1. Partial molecular structures of drug candidates A and B.
Fig. S2. Data analysis workflow.
Fig. S3. Diffusion of drug candidates into gelatin.
Fig. S4. ROI selection in four dog liver tissues.
Fig. S5. Calibration curves obtained in the multiplatform comparison.

Table S1. Intensity ratio and standard deviation of calibration points in multiplatform comparison

Calibration level	Concentration (µg/g)	Drug candidate A					
		Xevo Q-ToF	Synapt Q-ToF			Xevo QqQ	
		MS scan	MS scan	HDMS	HDMS^E	MS scan	MRM
C0	0.00	<LOD	<LOD	<LOD	<LOD	<LOD	<LOD
C1	12.50	0.01 ± 0.00	<LOD	<LOD	<LOD	<LOD	<LOD
C2	24.99	0.01 ± 0.00	<LOD	<LOD	<LOD	<LOD	<LOD
C3	124.97	0.02 ± 0.01	0.04 ± 0.02	<LOD	<LOD	<LOD	0.04 ± 0.02
C4	249.96	0.04 ± 0.02	0.09 ± 0.07	0.31 ± 0.36	<LOD	<LOD	0.19 ± 0.14
C5	500.11	0.15 ± 0.12	0.34 ± 0.30	1.26 ± 0.42	1.56 ± 0.66	0.13 ± 0.03	0.43 ± 0.20
C6	1250.08	0.53 ± 0.05	0.98 ± 0.36	3.34 ± 0.32	2.89 ± 0.06	0.26 ± 0.05	1.90 ± 0.51
C7	2500.14	1.19 ± 0.09	1.56 ± 0.28	8.46 ± 2.98	7.43 ± 2.97	0.55 ± 0.11	3.40 ± 0.27
		n=3	*n=3*	*n=3*	*n=3*	*n=3*	*n=3*

Calibration level	Concentration (µg/g)	Drug candidate B					
		Xevo Q-ToF	Synapt Q-ToF			Xevo QqQ	
		MS scan	MS scan	HDMS	HDMS^E	MS scan	MRM
C0	0.00	<LOD	<LOD	<LOD	<LOD	<LOD	<LOD
C1	12.50	0.02 ± 0.01	0.07 ± 0.02	0.22 ± 0.36	<LOD	<LOD	0.49 ± 0.04
C2	24.99	0.04 ± 0.02	0.30 ± 0.32	0.52 ± 0.56	<LOD	<LOD	2.09 ± 1.55
C3	124.97	0.14 ± 0.07	0.30 ± 0.27	0.71 ± 0.53	1.30 ± 1.08	0.23 ± 0.09	7.90 ± 6.00
C4	249.96	0.33 ± 0.12	0.69 ± 0.28	2.02 ± 1.20	3.78 ± 1.64	0.21 ± 0.16	13.30 ± 7.19
C5	500.11	1.26 ± 0.88	8.59 ± 1.06	67.13 ± 24.30	46.85 ± 48.27	0.41 ± 0.27	28.98 ± 5.52
C6	1250.08	6.20 ± 0.51	16.04 ± 3.09	146.86 ± 97.52	162.61 ± 56.86	1.24 ± 0.32	73.34 ± 10.34
C7	2500.14	13.45 ± 0.72	29.00 ± 12.03	318.08 ± 6.31	372.50 ± 175.60	2.59 ± 0.25	143.67 ± 19.12
		n=3	*n=3*	*n=3*	*n=3*	*n=3*	*n=3*

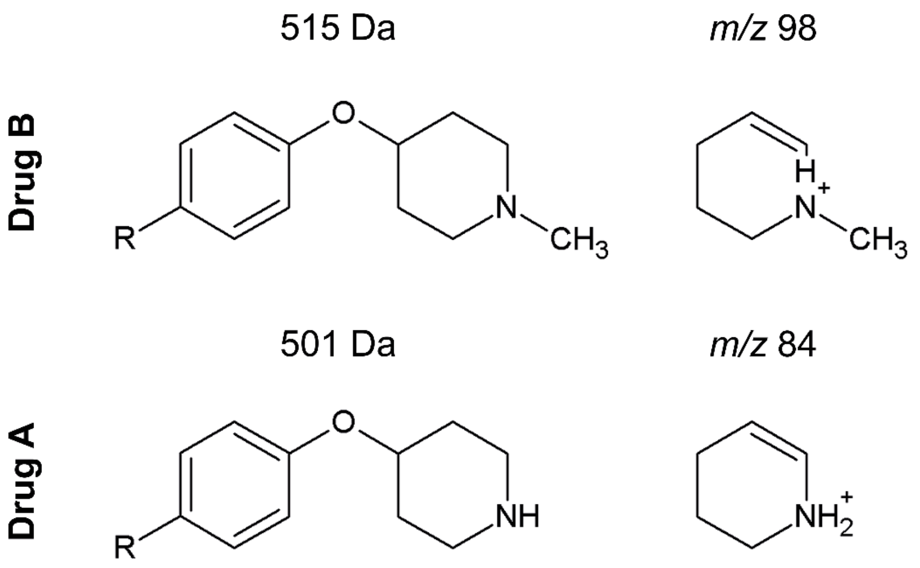

Fig. S1. *Partial molecular structures of drug candidate A and B at, respectively, 501 and 515 dalton. Two most-likely fragment ions that are used in the MRM transitions are shown at m/z 98 and m/z 84.*

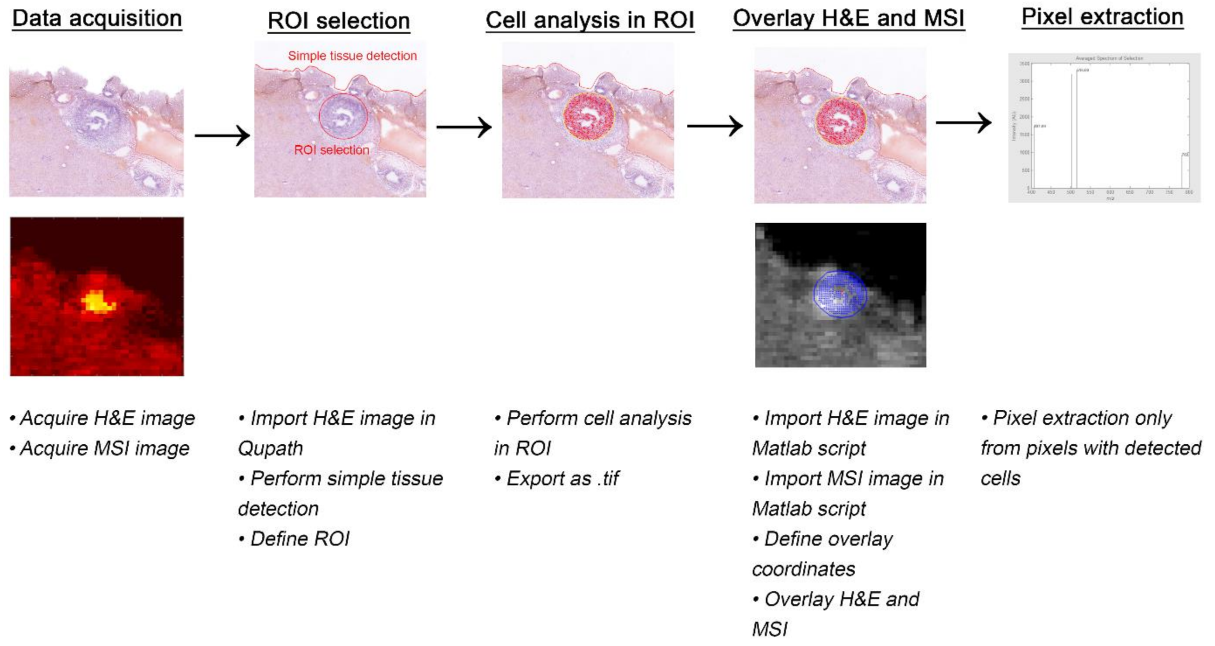

Fig. S2. *Data analysis workflow consists of a 5-step procedure. Step 1: MSI and H&E images are acquired. Step 2: ROI selection in Qupath. Step 3: Cell analysis in Qupath to detect cells. Step 4: Overlay H&E image (with detected cells marked) and MSI image in in-house written Matlab script. Step 5: MSI pixel selection and extraction in in-house written Matlab script. From these MS spectra, intensity ratios between the drug candidates and endogenous lipid are used.*

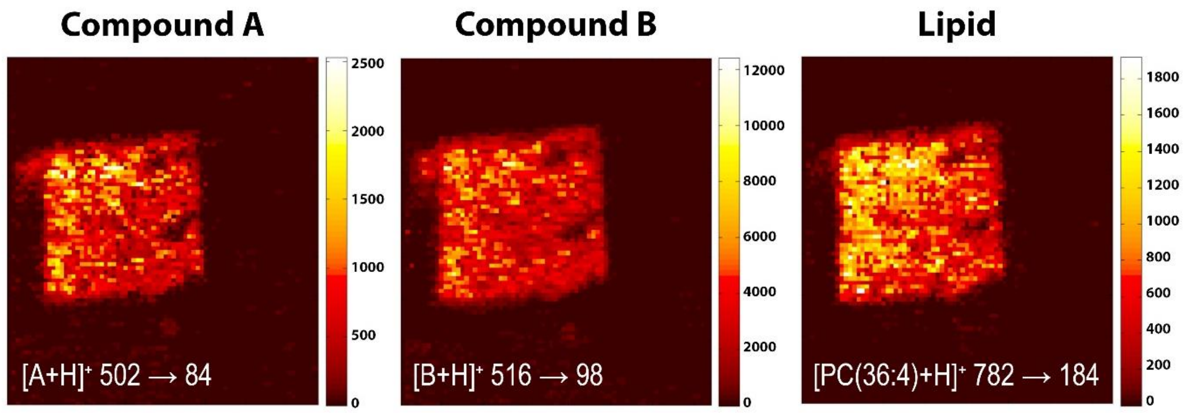

Fig. S3. *Diffusion of drug candidates into gelatin is shown by DESI-MRM images of a tissue homogenate pillar for drug candidate A, drug candidate B, and the endogenous lipid (used as a correction factor). The MRM images were acquired in positive ion mode at a spatial resolution of 50 µm. Dwell times were 0.247 s/pixel.*

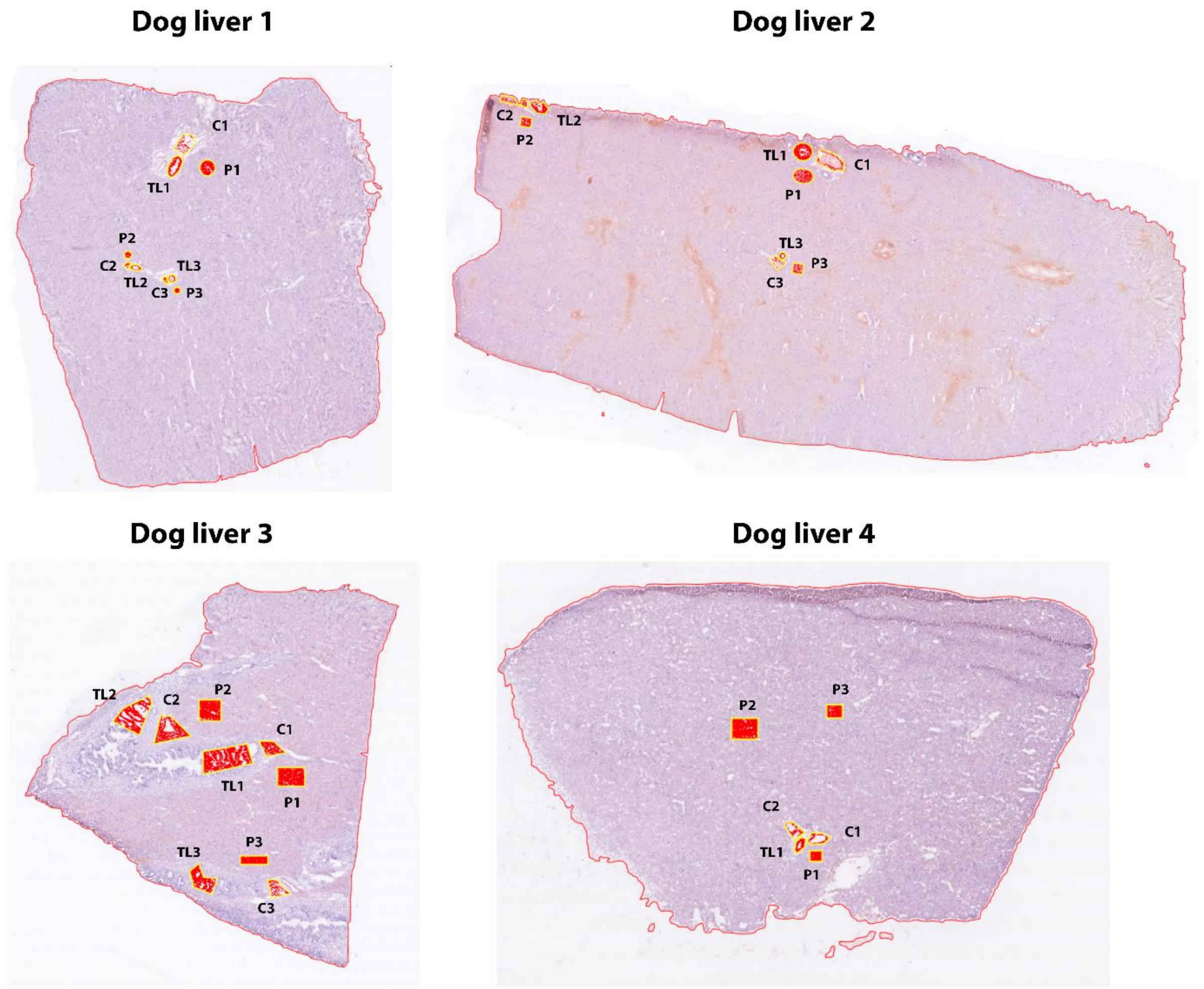

Fig. S4. *ROI selection in four dog liver tissues in their H&E images (Qupath software). For each dog liver tissue, the tissue is annotated by a pathologist and classified as tissue lesion (TL), connective tissue (C), and parenchyma (P). Where possible for each dog liver, ROIs of all tissue types are extracted in triplicate (f.e. TL1, TL2, and TL3). These H&E images were overlaid with the MSI images as described in Fig. S1.*

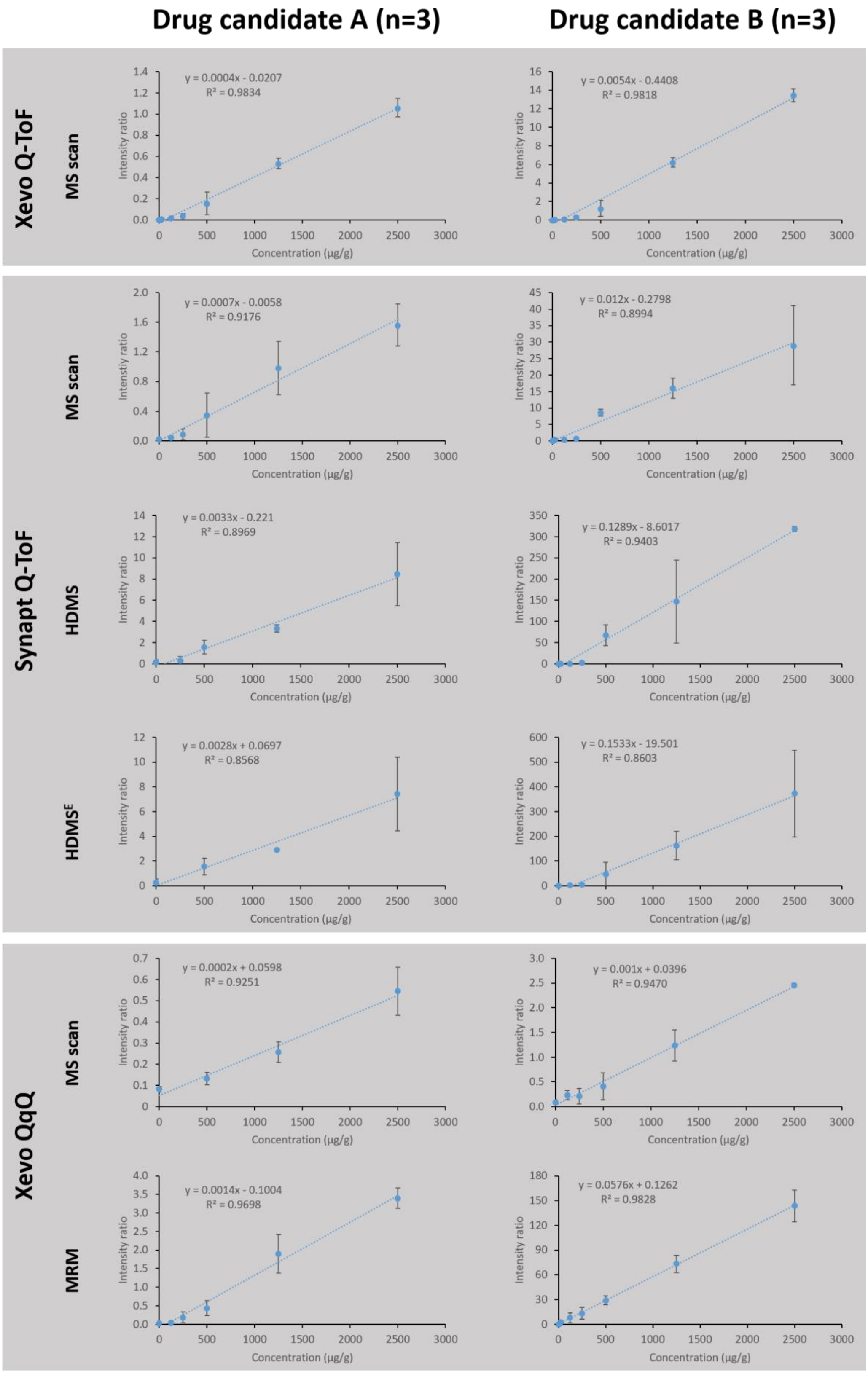

Fig. S5. *Calibration curves obtained in the multiplatform comparison for drug candidates A (left column) and B (right column). The multiplatform comparison includes calibration lines achieved on the Xevo Q-ToF (MS scan), Synapt Q-ToF (MS scan, HDMS, and HDMS^E), and Xevo QqQ (MS scan and MRM). Error bars show the standard deviation of the intensity ratio for each calibration level. This figure complements Table 1 and Table S1.*
